# Cardiovascular anesthesia training: A single center survey among fellow doctors

**DOI:** 10.1097/MD.0000000000035570

**Published:** 2023-11-10

**Authors:** Yan-Ting Sun, Wei Wu, Jian-Qun Liang, Yun-Tai Yao

**Affiliations:** a Department of Anesthesiology, Baoji High-Tech Hospital, Shaanxi, China; b Department of Anesthesiology, Fuwai Hospital, National Center for Cardiovascular Diseases, Peking Union Medical College and Chinese Academy of Medical Sciences, Beijing, China; c Department of Digestive Endoscopy Center, Baoji High-Tech Hospital, Shaanxi, China.

**Keywords:** cardiovascular anesthesiology, fellow, refresher training

## Abstract

Despite the proliferation of research on anesthesiology training at all stages of medical education, there is relatively little published literature surveying the perspectives and concerns of anesthesiologists regarding cardiovascular anesthesia training. Therefore, we conducted a survey to investigate the attitudes, barriers, expectations, stress experiences, satisfaction, and future aspirations of anesthesiologists trained at a tertiary cardiovascular specialty hospital in China. A questionnaire survey was conducted among 260 anesthesiologists who received cardiovascular anesthesia training at departments of anesthesiology in a tertiary cardiovascular specialty hospital in China. After the study protocol was approved, electronic questionnaires were distributed to the target group through the online survey software “Wen Juan Xing.” Respondents were asked to complete an anonymous questionnaire on their smartphones through WeChat, with the restriction of one response per device enabled. Of the 260 trainees, 240 (98%) completed the questionnaire. The majority of the trainees were 31 years of age or above. A large majority had approximately 10 years of clinical anesthesia practice, and nearly one-third had never undertaken cardiovascular specialty anesthesia practice before. The most common reasons for attending the refresher training were the need to learn basic specialty theory and improve clinical skills. The barriers were mainly time constraints or staff shortages in the department. Sixty-one (93.8%) trainees described the experience as “stressful or highly stressful” and identified poor teacher interaction as the highest-ranking stressor. Anesthesiologists were most dissatisfied with job rewards, with a satisfaction rate of only 15%. Anesthesiologists are highly stressed during the refresher training. Poor teacher interaction and low job rewards were identified as the highest-ranking stressors during cardiovascular anesthesia training. Training providers need to pay more attention to these stressors to enhance the quality of cardiovascular anesthesia training.

## 1. Introduction

The time-honored strategy by which anesthesiologists update their knowledge and skills is through continuing medical education (CME).^[1–3]^ In China, CME encompasses standardized residency training, postgraduate education, and refresher training. Residency training for all physicians, including anesthesiologists, is becoming increasingly standardized. The mandated model is a “5 + 3” plan, which has been significantly supported by our government through substantial monetary investments.^[4]^ However, refresher training in cardiovascular anesthesiology is not yet standardized and varies between hospitals.

Currently, there is no accredited body to certify cardiac anesthesiologists or oversee specialty cardiovascular anesthesiology training in China.^[5,6]^ Most Chinese cardiovascular anesthesiologists receive specialized training from departments of anesthesiology at major cardiac hospitals. Fuwai Hospital, the National Center for Cardiovascular Disease in China and one of the world largest cardiovascular centers, has been enrolling cardiovascular anesthesia fellow physicians since 1956. Annually, 40–60 fellow doctors from hospitals around China undertake half or 1-year specialty training programs in cardiovascular anesthesia at Fuwai Hospital.^[7]^

To optimize the utilization of educational content, a better understanding of the appropriate educational activities that will meet the needs of trainees is necessary.^[8,9]^ Therefore, we conducted a descriptive cross-sectional survey to investigate the attitudes, barriers, expectations, and preferred learning models of content and approach towards refresher training. Moreover, we explored the satisfaction and stress levels experienced by anesthesiologists who trained at a tertiary cardiovascular specialty hospital in China. This research may assist policymakers and training providers in tailoring their strategies to comprehensively improve the quality of training services, to meet learners’ needs, optimize the overall learning experience, and contribute to the broader literature.

## 2. Methods

This study was a regional, internet-based, cross-sectional survey, and all the participants provided informed consent. After receiving approval for the need for informed consent from the Ethical Committee of Fuwai Hospital, data were collected from October 2022 to December 2022. The inclusion criteria were as follows: fellow doctors who have received cardiovascular anesthesia training from the departments of anesthesiology at Fuwai Hospital since the onset of the COVID-19 pandemic in January 2020. All study procedures, protocols, and methods were performed in accordance with relevant guidelines and regulations.

### 2.1. Study design and instrument development

A comprehensive 52-item questionnaire was constructed, comprising 16 open-ended questions, 31 single-choice, and 5 multiple-choice questions (see Table S1, http://links.lww.com/MD/K444). The draft questionnaire was designed based on group discussions and a literature review. Furthermore, to ensure the incorporation of critical issues associated with cardiovascular anesthesia training, a pilot study was conducted with 20 trainees from 5 provinces, both with and without previous cardiovascular anesthesia experience (see Acknowledgements). The final version of the electronic questionnaire was developed using the online survey software “Wen Juan Xing” (wjx.sojump.com) and was disseminated to anesthesiologists who had undergone specialty training at the departments of anesthesiology at Fuwai Hospital via WeChat, with the restriction of one response per device enabled.

### 2.2. Data collection

Trainees were asked about their attitudes toward and perceived restrictions with the training, as well as, their motivations, expectations, preferred content and approaches, cost and financial compensation, stress experiences, stressors, and potential solutions to stress. The inquiries regarding satisfaction with the training encompassed aspects such as pre-training orientation, video-based curriculum, admission procedure, teaching methodology, scheduling, and financial compensation. Stress levels were gauged using a 4-point Likert scale,^[10]^ where 1 = strongly stressful, 2 = stressful, 3 = not stressful, 4 = not stressful at all. Satisfaction items were ranked on a similar 4-point Likert scale (1 = strongly satisfied, 2 = satisfied, 3 = dissatisfied, 4 = strongly dissatisfied). Of the 52 survey questions, 16 were open-ended, exploring the concerns of fellow doctors in various aspects including reasons, expectations, and demands, suggestions for managers and teachers, feedback on the video-based curriculum, and perceived regrets at the end of the training.

Additional variables addressing the social demographics of trainees were collected, including sex, name, age, professional title, ethnicity, marital status, whether they had children, educational background, hospital grade, annual cardiovascular surgical volume, and years of experience in clinical anesthesia and cardiovascular specialty anesthesia practice.

### 2.3. Data analysis

All data were stored and analyzed using Microsoft Excel 2007. GraphPad Prism software (version 8.0) was employed to generate figures. The results are presented as absolute numbers and percentages (%). Responses to open-ended questions were consolidated into 3 overarching themes. After identifying overlapping answers, these themes were then categorized into 7 distinct categories, with representative comments illustrating each category. This process was iteratively conducted by 2 authors (Y.T.S. and W.W.). Answer reduction was executed by YTS and JQL and cross-verified by YTY.

## 3. Results

### 3.1. Social demographics

**Figure 1.**
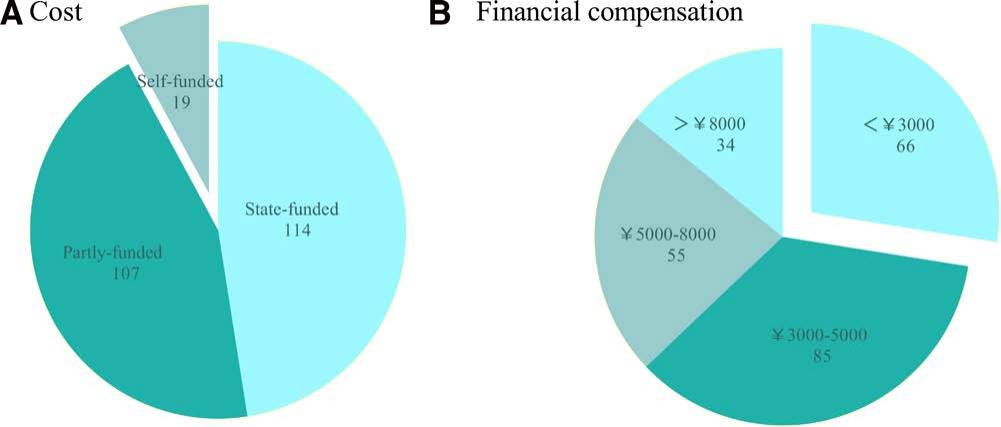
Cost (A) and financial compensation (B) of fellow doctors when pursuing cardiovascular anesthesia training.

We received 240 eligible responses with a response rate of 92.3%. Twenty responses were discarded as they were identified as incomplete or inappropriate. The median time to complete the survey was 9.5 minutes, with an interquartile range (IQR) of 6.5 to 12.3 minutes. Anesthesiologists from more than 8 other hospitals throughout China (Henan, Hebei, Shandong, Anhui, Shanxi, Sichuan, and Yunnan) received cardiovascular anesthesia training at Fuwai Hospital. For 87.6% of respondents, the training lasted for half a year, and 12.3% received 1 year of cardiovascular anesthesia training. As shown in Table 1, the majority of trainees were aged 31 and above and 122 (50.8%) were male. Pertaining to educational qualifications, half of the trainees possessed master degrees, were employed at tertiary general hospitals, and held positions at the level of attending physician or higher. A substantial portion of the respondents were married (84.6%) and were parents to either one (37.1%) or 2 (44.6%) children. Notably, a large majority (90.8%) had accrued over 5 years of experience in clinical anesthesia practice, yet almost a third had not previously engaged in cardiovascular specialty anesthesia practice. Concerning financial aspects, over half of the respondents financed or partially financed their training fees, receiving a financial compensation less than 5000 RMB during the training period (as illustrated in Fig. 1)

**Table 1:** Respondents’ social demographics data (N = 240).

Variable	Responses	Number of participants, N (%)
① Sex	Male	122 (50.8)
	Female	118 (49.2)
② Age range	25–30 yr	18 (7.5)
	31–35 yr	118 (49.2)
	36–40 yr	89 (37.1)
	41 yr and above	15 (6.3)
③ Ethnicity	Han	214 (89.2)
	Others	26 (10.8)
④ Professional title	Resident	18 (7.5)
	Attending physician	188 (78.3)
	Associate professor	34 (14.2)
	Professor	0 (0)
⑤ Educational background	Bachelor degree	107 (44.6)
	Master degree	122 (50.8)
	Doctoral degree	11 (4.6)
⑦ Marital status	Single	30 (12.5)
	Married	203 (84.6)
	Divorced before starting refresher training	7 (2.9)
⑧ Having children, No.(n)	0	44 (18.3)
	1	89 (37.1)
	2	107 (44.6)
⑨ Years range of clinical anesthesia experience	3–5 yr	22 (9.2)
	6–10 yr	114 (47.5)
	10–15 yr	92 (38.3)
	16 yr and above	12 (5.0)
⑩ Years range of cardiovascular specialty anesthesia experience	0 yr	89 (37.1)
	0–2 yr	66 (27.5)
	3–5 yr	30 (12.5)
	5–10 yr	37 (15.4)
	11 yr and above	18 (7.5)
⑫ Province where respondents worked	Henan	30 (12.5)
	Hebei	26 (10.8)
	Shandong	26 (10.8)
	Anhui	18 (7.5)
	Shanxi	15 (6.3)
	Sichuan	11 (4.6)
	Yunnan	11 (4.6)
	Other	103 (42.9)
⑬ Grade of the hospital	Second hospital	4 (1.7)
	Tertiary hospital	236 (98.3)
⑮ Clinical practice setting	General hospital	229 (95.4)
	Specialty hospital	11 (4.6)
⑯ Annual cardiovascular surgical volume. Patient. No.(n)	0	44 (18.3)
	1–50	18 (7.5)
	51–200	74 (30.8)
	201–500	52 (21.7)
	501–1000	18 (7.5)
	1001–2000	15 (6.3)
	2001–3000	11 (4.6)
	3000 and above	8 (3.3)

① represents the first question of the questionnaire, and so on. Results were presented as absolute number percentage (%).

### 3.2. Status of refresher training

As highlighted in Table 2, the majority of trainees rarely returned home during their training stint. A considerable proportion (two-thirds) exhibited a strong or very strong positive inclination towards attending the cardiovascular specialty training at Fuwai Hospital. Moreover, 78.3% expressed a preference for instructors holding the title of associate director or a higher designation during the training period. A preference for permanent instructors was echoed by over half of the respondents. When questioned about recommending Fuwai Hospital for refresher training to colleagues, a substantial 78.3% (188 individuals) affirmed their willingness to recommend or strongly recommend it. The primary impediments cited for attending refresher training were time constraints (46.3%) and staffing shortages in their respective departments (30.4%), followed by insufficient financial compensation (17.1%) and disagreements with authorities (6.3%).

**Table 2:** Refresher training data (N = 240).

Variable	Responses	Number of participants, N (%)
㉒ Attitude towards refresher training	Strongly positive	118 (49.2)
	Positive	100 (41.7)
	Dispositive	0 (0)
	Strongly dispositive	22 (9.2)
⑲ Motivation of attending refresher training	Updating clinical technical skills, knowledge, and competencies	229 (95.4)
	Title needs	122 (50.8)
	Improvement for future job opportunities	81 (33.8)
	The hospital is going to perform heart surgery	7 (2.9)
	Career improvement for current job	4 (1.7)
⑳ Restrictions respondents perceived in the training	Time constraint	111 (46.3)
	Financial compensation	41 (17.1)
	The authority disagreed	15 (6.3)
	Shortage of staff in the department	73 (30.4)
㊶ Preferred to be taught by a teacher?	Resident	0 (0)
	Attending physician	33 (13.8)
	Associate professor	155 (64.6)
	Professor	33 (13.8)
	whatever	19 (7.9)
㊸ Are you willing to recommend other colleagues for refresher training at Fuwai Hospital?	Strongly recommend	106 (44.2)
	Recommend	82 (34.2)
	Not recommend	48 (20.0)
	Strongly not recommend	4 (1.6)
㊵ Do you want the teacher to be permanent?	Yes	125 (52.1)
	No	63 (26.3)
	Whatever	52 (21.7)
㉖ Times of the trainees went home during the refresher training.	0	70 (29.2)
	1–3	137 (57.1)
	3–5	22 (9.2)
	>5	11 (4.6)
㉗ Days of the trainees went home during there fresher training.	0	89 (37.1)
	1–5	55 (22.9)
	6–10	85 (35.4)
	11–15	11 (4.6)
	>15	18 (7.5)
㊳ What you have improved through refresher training?	Clinical knowledge of cardiovascular anesthesia	111 (46.3)
	Clinical technical competence	210 (87.5)
	Perioperative management	207 (86.3)
	Research ability	37 (15.4)

### 3.3. Stress and satisfaction

**Figure 2.**
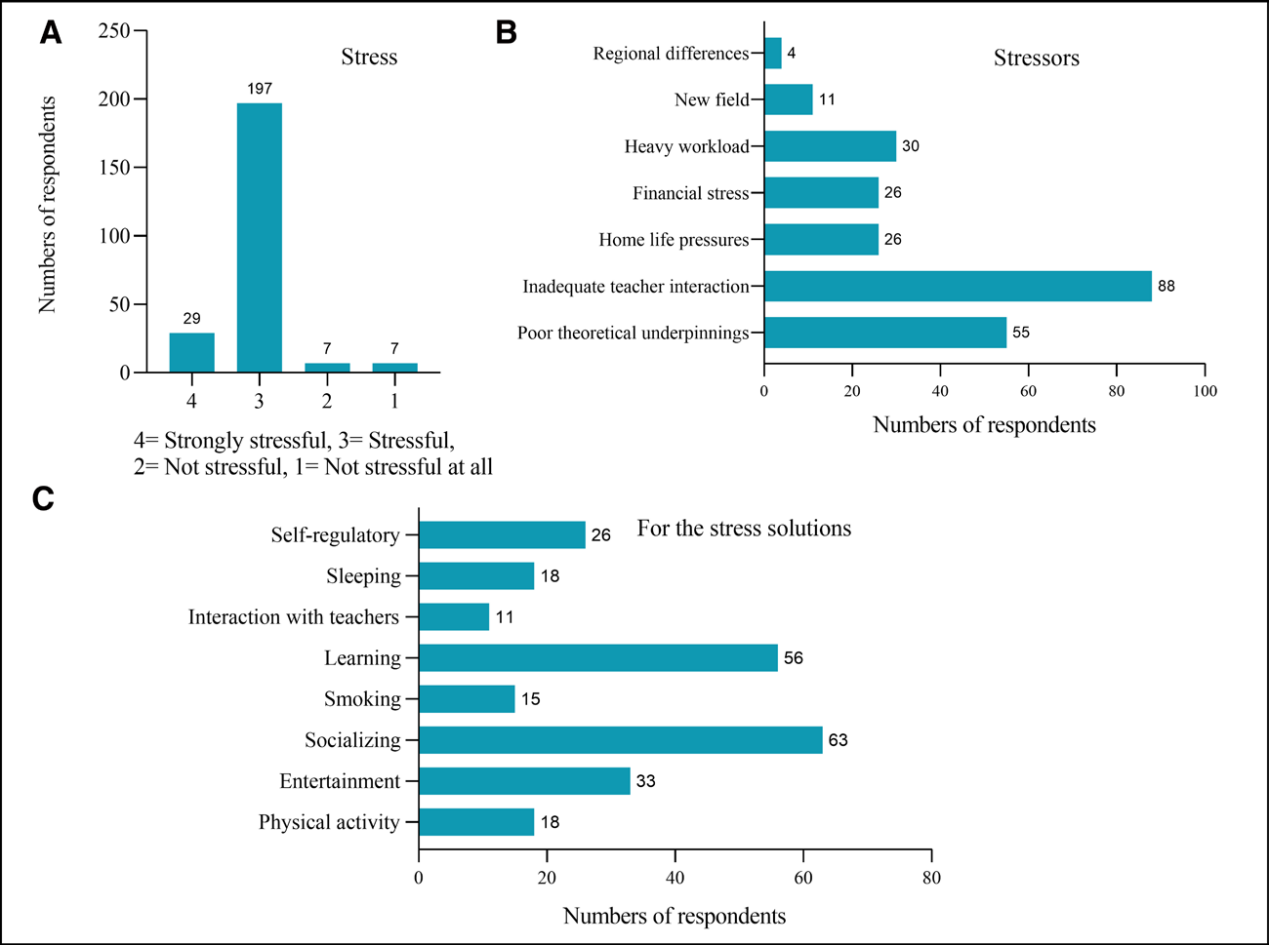
Trainees’ stress experience (A), stressors (B), for the stress solutions (C).

**Figure 3.**
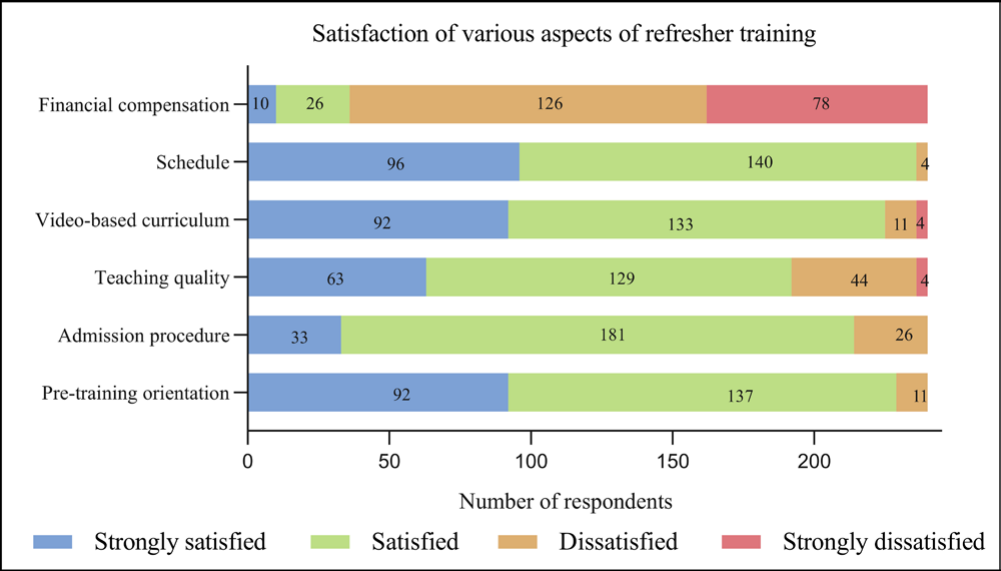
Satisfaction of various aspects of refresher training at a tertiary academic hospital in our sample confirmed including orientation before training, admission procedure, teaching, video-based curriculum, schedule, and financial compensation, respectively.

Two hundred twenty-six (94.2%) trainees indicated that their experience was either “stressful” or “highly stressful” (Fig. 2A) and identified 7 major sources of training stress: poor theoretical underpinnings, inadequate teacher interaction, home life pressures, financial stress, heavy workload, adjusting to a new field, and regional differences (Fig. 2B). When surveyed about potential stress alleviation strategies (such as physical activity, entertainment, socializing, smoking, learning, interaction with teachers, sleeping, and self-regulation) (Fig. 2C), the trainees ranked social activities and learning as the most useful. Figure 3 displays the responses to the satisfaction survey, with the following percentages for satisfaction or strong satisfaction: 95% for pre-training orientation, 89% for the admission procedure, 80% for teaching quality, 94% for the video-based curriculum, 98% for the schedule, and 15% for financial compensation, respectively.

### 3.4. Open-ended questions

As shown in Table 3, the trainees chose a tertiary academic hospital for their studies mainly due to its specialization, a large volume of clinical cardiovascular and surgical cases, and the hospital strong reputation. The reasons given by the trainees for attending refresher training varied widely. Most respondents preferred learning modes including interpersonal interaction, workshops, and integration of theory with practice.

**Table 3:** Open-ended questions data.

		Responses	N (%)
		Large number of clinical cardiovascular surgical cases	41 (17.1)
		Specialization	37 (15.4)
		Hospital reputation	41 (17.1)
	Category-1 Reasons for refresher training at Fuwai Hospital	Short distance	4 (1.7)
		Development needs of hospital	30 (12.5)
		For the title needs	11 (4.6)
		Overall consideration (e.g., learn basic specialty theory, improve clinical skills; increase anesthesia experience; study perioperative management of cardiac surgery; go back to original department to be able to solve difficult problems; broaden the horizons)	64 (26.7)
Themes1: Before refresher training	Category-2 Expectations about the content of the study before the engage in refresher training.	Hear from and interact with the specialist, be proficient in anesthesia pathophysiology and corresponding clinical treatment of various cardiovascular operations, play an independent role in cardiovascular anesthesia operation after refresher training	79 (32.9)
		Learn more about electrocardiograph	4 (1.7)
		Learn the application of vasoactive drugs and understand the pathophysiology of heart disease	71 (29.6)
		Clinical teachers can give more guidance through interpersonal interaction, hands-on practice, problem-based learning, simulation education, and conceptual-practice integration	38 (15.8)
		Transesophageal echocardiography	4 (1.7)
		Familiar with monitoring techniques	8 (3.1)
		Self sublimation	19 (7.9)
	Category-3 Demands during training at Fuwai Hospital.	The leading teacher patience and guidance (enhance the awareness of teaching)	71 (29.6)
		Humanistic care (feedback of learning, sense of belonging, there are holidays home during the study period, happy learning)	34 (14.2)
		Accommodation, allowance	41 (17.1)
		Schedule personal study time	19 (7.9)
		Development an appropriate short curriculum specialty for refresher training	75 (31.3)
		Research ability training	4 (1.7)
	Category-4 Suggestions for managers	Training for anesthesia trainers to improve the knowledge and skills	49 (20.4)
		Development or modification of an appropriate short curriculum specialty for refresher training	49 (20.4)
		Establishing effective ongoing monitoring, feedback, and evaluation tool	75 (31.3)
		Scientific research and teaching	8 (3.3)
		Adequate funding support	38 (15.8)
Themes2: After refresher training	Category-5 Suggestions for teachers	More teacher interaction	75 (31.3)
		A teacher training and emphasizing early handover of teaching to local instructors may be needed	34 (14.2)
		Teaching by conceptual-practice integration rather than only practice	94 (39.2)
		To be taught by a teacher with the title of associate director or higher	26 (10.8)
	Category-6 Suggestions for educational curriculum	Arrange time reasonably; there are special teaching hours for fellows	86 (35.8)
		Develop a curriculum and teaching materials instructional design specifically for refresher training	56 (23.3)
		Provide multiple teaching techniques including problem-based learning, case discussion, and simulation education	98 (40.8)
		Failed to achieve the purpose of refresher training (did not learn more about related departments, had no systematic knowledge of cardiovascular theory, the types of anesthesia for cardiac surgery were not studied comprehensively, the type of surgery exposure was not comprehensive)	60 (25.0)
		Didn’t follow a lot of teachers	15 (6.3)
Themes3: regrets	Category-7: Regrets perceived at the end of the training	There were too few group activities in the department	8 (3.3)
		The time for refresher training was too short	11 (4.6)
		Failed to enter transesophageal echocardiography	11 (4.6)
		Failed to enter the clinical research	3 (1.3)

## 4. Discussion

This study presents critical information regarding the current state of cardiovascular anesthesia training among anesthesiologists in China. The findings highlight a significant level of stress experienced by anesthesiologists during their refresher training, with inadequate teacher interaction emerging as the highest-ranking stressor. Furthermore, there is a notable level of dissatisfaction concerning job rewards, evidenced by a mere 15% satisfaction rate. A majority of the trainees bear the brunt of the training expenses either partially or in full, and given that many have families with 1 or 2 children, financial compensation stands as a significant barrier, diminishing their willingness to participate in the training. It is imperative that training providers turn their attention to these stressors to enhance the quality of cardiovascular anesthesia training in the country. These insights may spur concerns about the current state of cardiovascular anesthesia refresher training in China, potentially encouraging the government to amplify financial backing for such programs.

### 4.1. Characteristics of fellow doctors

Refresher training, in a CME format, has its own characteristics. According to our survey, the fellow doctors participating in this training span a wide age range, with the majority holding a master or even a doctoral degree, and occupying positions at the attending level or higher. Moreover, many possess around a decade of clinical work experience. This diverse background in basic professional theories and clinical experience often results in a lack of standardization when it comes to clinical disease diagnosis, surgical operations, and perioperative management, posing challenges in the overall management and necessitating a diversified approach in designing refresher training content and formats. Furthermore, the motivations behind attending refresher training were varied amongst our trainees. While some sought to enhance their foundational specialty theory knowledge, hone clinical skills, and accumulate surgical experience, others participated solely to fulfill the title requirements that necessitated completing the training. Additionally, a portion were already in departmental leadership roles and were focused on specialized disease and operation studies. A few chose refresher training to meet the developmental needs of their hospitals. Harrison et al^[8]^ noted that the primary motivations for attending refresher training were to update knowledge, gain reassurance that their practices were in line with current guidelines, and to engage with specialists in the field. Furthermore, for a variety of reasons, fellow doctors generally undertook half a year to 1 year of cardiovascular anesthesia training, with a consensus leaning towards the half-year mark as the optimal training duration. According to the World Federation of Societies of Anesthesiologists Global Anesthesia Workforce Survey,^[11]^ the training duration for specialist anesthesiologists varied greatly, ranging from less than a year to over 5 years. This data underscores a potential inconsistency and lack of standardization among training providers within countries, with some offering short-term or informal training programs lacking a defined curriculum or structured approach.

### 4.2. Attitudes and restrictions towards refresher training

A substantial majority of trainees already had approximately a decade of clinical anesthesia practice under their belts. Despite this extensive experience, they exhibited a strong or very strong positive attitude towards attending cardiovascular specialty training. They were keen to explore the perspectives and experiences of others, seek advice, compare notes, exchange ideas, delve into the latest research, and appreciate and assimilate the viewpoints of peers to further hone their clinical technical skills and elevate their academic standing. Regarding perceived barriers to training, time constraints and staffing shortages in their respective departments were most commonly cited, followed by issues of financial compensation and disagreements at the authority level. The demanding schedules of anesthesiologists have resulted in limited opportunities to participate in refresher training. The issue of facilitating CME in time-scarce environments has persisted at all tiers of medical training for many years.^[1,12]^ Currently, there are not enough anesthesia providers to satisfy basic clinical demands, let alone spare the manpower for refresher training in the face of departmental staff shortages.

### 4.3. Stress and satisfaction

One important finding was that poor teacher interaction was the highest scoring stressors followed by poor theoretical expertise and workload. A tertiary academic hospital, where hospitals are overloaded, anesthesiologists are overworked, and for the few involved in teaching, most time is taken up by clinical issues and administrative.^[4]^ This imbalance between a sparse healthcare workforce and an escalating caseload is a recurring theme in many teaching hospitals. According to the Chinese Society of Extra-Corporeal Circulation statistics, 734 hospitals reported 207,781 cardiac surgery cases in 2013, and 209,765 cases in 2014. A tertiary academic hospital performed a total cardiac surgery volume of 13,755 in 2015, with 209,765 in mainland China.^[4]^ Anesthesia providers in places that need to train people do not have the time or resources to do so. In these environments, external support may be crucial for developing refresher training programs, such as emphasizing handover of teaching to local instructors and teacher training.^[13,14]^ Despite these challenges, we noted a high level of satisfaction among respondents with the leadership and instructional quality at tertiary academic hospitals regarding the content offered.

### 4.4. Teaching and learning

Due to the COVID-19 global pandemic, web-based platforms for video-based curricula are being utilized as substitutes for in-person curricula to offer educational support.^[9]^ A tertiary academic hospital is providing video conferencing educational opportunities during the early mornings of working days for residents, fellow physicians, and graduate students. However, the schedule of these courses conflicts with the duty hours of fellow doctors. Additionally, there is no course content or teaching model specifically designed for refresher training. To address this, specific support, including the development of a curriculum and instructional design exclusively tailored for refresher training, is required. This might entail establishing training programs and fostering local clinical teaching initiatives. According to survey data, most respondents favor learning modes that include interpersonal interaction (such as formal and informal discussions with colleagues), hands-on practice (like workshops), problem-based learning, simulation education, and the integration of theory with practice. Case discussions^[15]^ enable learners to solidify their knowledge, skills, and understanding of nuanced details through the experiences of others. Problem-based learning^[16]^ helps students to cultivate problem-solving capabilities while simultaneously enhancing their foundational and clinical knowledge and skills. Simulation education, facilitated by training practice scenarios, offers a safe, interactive, efficient, and engaging method of teaching. While these learning modes have proven to be effective, their potential would be considerably diminished without structured tools for periodic assessment, leading to the possible wastage of limited resources. Utilizing a mix of qualitative and quantitative surveys to gather feedback from trainees is the optimal strategy for evaluating the quality of training and fostering further development.

### 4.5. Suggestions

Regardless of the anesthesia provision model, excellent refresher training must be finely tuned to meet the needs of trainees. To do this, the development of successful refresher training programs requires, like other education cases,^[17–19]^ the following 1, Support for training programs at all levels, including regional governments, professional medical organizations, and the institutions where fellow doctors are employed; 2, Adequate financial backing for both trainers and trainees; 3, The development or modification of a specialized short curriculum specifically for refresher training; 4, Establishing effective ongoing monitoring, feedback, and evaluation tools; 5, The delegation of teaching responsibilities to qualified local instructors, as opposed to relying on overworked anesthesiologists who are doing their best under difficult circumstances; 6, and Training initiatives for anesthesia trainers aimed at enhancing both technical and non-technical skills (such as leadership, research, and communication), which will be instrumental in driving the development of the relevant specialty.

## 5. Limitations

To accurately interpret the results of our study, it is essential to acknowledge several limitations: The study was conducted as a regional, internet-based, cross-sectional survey, which inherently has certain limitations. Potential biases associated with such surveys include non-response bias, agreement bias, and social desirability bias. The study primarily encapsulates the experiences of anesthesiologists who trained at a tertiary cardiovascular specialty hospital in China. This might limit the generalizability of the findings. However, we sought to minimize selection bias by only enrolling fellow doctors who have joined since the onset of the COVID-19 pandemic in January 2020. Due to the nature of the data distribution and categorization, the results are presented in terms of absolute percentages (%). Consequently, the scope of statistical analysis was restricted.

## 6. Conclusion

This study contributes valuable insights to the existing body of literature on the perspectives and challenges faced by anesthesiologists during cardiovascular anesthesia training in China. The findings indicate that anesthesiologists experience significant stress during refresher training, with poor teacher interaction and inadequate job rewards emerging as the primary sources of stress. However, more efforts are needed from training providers to guarantee high-quality refresher training. We anticipate that these findings will serve to enlighten and encourage reflection among regional governments, professional medical organizations, and institutions where fellow doctors are employed, thereby enriching the broader literature.

## Author contributions

**Conceptualization:** Yan-Ting Sun.

**Investigation:** Jian-Qun Liang.

**Methodology:** Yun-Tai Yao.

**Supervision:** Yun-Tai Yao.

**Writing – original draft:** Yan-Ting Sun.

**Writing – review & editing:** Wei Wu, Yun-Tai Yao, Jian-Qun Liang.

## Supplementary Material


