# Supplementary material for: Cardiovascular anesthesia training: A single center survey among fellow doctors
Source: Medicine (Baltimore). 2023 Nov 10;102(45):e35570. doi: 10.1097/MD.0000000000035570 (PMC10637559; doi:10.1097/MD.0000000000035570)
Supplement: Supplementary file 1 [file medi-102-e35570-s001.docx]

| Table S1. Questionnaire for fellows who trained since the COVID-19 pandemic began in January 2020 at Fuwai hospital | | |
| --- | --- | --- |
| Question number | Question | Responses |
| Q.1 | Sex | 1. Male; 2. Female |
| Q.2 | Age | 1.＜25 years; 2. 25-30years; 3. 31-35years; 4. 36-40 years; 5. 41-50 years; 6. ≥50 years |
| Q.3 | Ethnicity | 1. Han; 2. Ethnic minority |
| Q.4 | Professional title | 1. Resident; 2. Attending physician; 3. Associate professor; 4. Professor |
| Q.5 | Educational background | 1. Bachelor’s degree; 2. Master’s degree; 3. Doctoral degree |
| Q.6 | Blood type | 1. O; 2. A; 3. B; 4. AB |
| Q.7 | Marital status | 1. Single; 2. Married; 3. Divorced before starting refresher training |
| Q.8 | Having children, No.(n) | 1. 0; 2. 1; 3. 2; 4. 3; 5. 4 |
| Q.9 | Years range of clinical anaesthesia experience | 1. 3-5 years; 2. 6-10 years; 3. 10-15years; 4. 16 years and above |
| Q.10 | Years range of cardiovascular specialty anaesthesia experience | 1. 0 years; 2. 1-2 years; 3. 3-5 years; 4. 6-10 years; 5. 11 years and above |
| Q.11 | Zodiac signs |  |
| Q.12 | Province where respondents worked | 1. Henan; 2. Hebei; 3. Shandong; 4. Anhui; 5. Shanxi; 6. Other ( please be specific) |
| Q.13 | Grade of the hospital | 1. Clinical practice setting; 2. Tertiary hospital |
| Q.14 | Where you works? (non-compulsory) |  |
| Q.15 | Clinical practice setting | 1. General hospital; 2. Specialty hospital |
| Q.16 | Annual cardiovascular surgical volume. Patient. No.(n) | 1. 0; 2. 1-50; 3. 51-200; 4. 201-500; 5. 501-1000; 6. 1001-2000; 7. 2000 and above |
| Q.17 | Cardiovascular surgery performed  in your department (multiple-choice question) | 1. Coronary intervention; 2. Valvular intervention; 3. Major vascular surgery; 4. Tumor; 5. Congenital heart disease |
| Q.18 | Special perioperative monitoring techniques performed in you department (multiple-choice question) | 1. Near-infrared spectrum; 2. Bispectral index; 3. Pulmonary artery pressure; 4. Left atrial pressure monitoring; 5. Transoesophageal echocardiography; 6. Others ( please be specific) |
| Q.19 | Motivation of attending refresher training (multiple-choice question) | 1. Updating clinical technical skills, knowledge, and competencies; 2. Title needs; 3. Improvement for future job opportunities; 4. Career improvement for current job; 5. Others ( please be specific) |
| Q.20 | Restrictions respondents perceived in the training | 1. Time constraint; 2. Financial compensation; 3. The authority disagreed; 4. Shortage of staff in the department |
| Q.21 | What are the reasons for choosing which hospital to  go to for refresher training. (multiple-choice question) | 1. Specialization; 2. Hospital’s reputation; 3. With renowned experts; 4. Short distance; 5. large number of clinical cardiovascular and surgical cases; 6. Scientific research programs |
| Q.22 | Attitude towards refresher training | 1. Strongly positive; 2. Positive; 3. Dispositive; 4. Strongly dispositive |
| Q.23 | Expectations about the content of the training before the engage in refresher training (open-ended question) |  |
| Q.24 | Cost of cardiovascular anesthesia training. | 1. Self-funded; 2. Partly-funded; 3. State-funded |
| Q.25 | Financial compensation of fellow doctors when pursuing cardiovascular anesthesia training. | 1. ＜￥3000 ; 2. ￥3000-5000; 3. ￥5000-8000; 4. ＞￥8000 |
| Q.26 | Times of the trainees went home during the refresher training (open-ended question) |  |
| Q.27 | Days of the trainees went home during the refresher training (open-ended question) |  |
| Q.28 | Respondents’ stress experience | 1. Strongly stressful; 2. Stressful; 3. Not stressful; 4. Not stressful at all |
| Q.29 | Respondents’ stressors (open-ended question) |  |
| Q.30 | Respondents’ solutions for the stress (open-ended question) |  |
| Q.31 | Length of your training at Fuwai Hospital | 1. Half a year; 2. One year |
| Q.32 | Case volume attended during the refresher training (open-ended question) |  |
| Q.33 | On duty attended during the refresher training (open-ended question) |  |
| Q.34 | Video-based curriculum attended during the training (open-ended question) |  |
| Q.35 | Satisfaction of orientation before training | 1. Strongly satisfied; 2. Satisfied; 3. Dissatisfied; 4. Strongly dissatisfied |
| Q.36 | Satisfaction of admission procedure | 1. Strongly satisfied; 2. Satisfied; 3. Dissatisfied; 4. Strongly dissatisfied |
| Q.37 | Satisfaction of the teaching at Fuwai Hospital | 1. Strongly satisfied; 2. Satisfied; 3. Dissatisfied; 4. Strongly dissatisfied |
| Q.38 | What you have improved after the training ? (multiple-choice question) | 1. Clinical knowledge of cardiovascular anaesthesia; 2. Clinical technical competence; 3. Perioperative management; 4. Research ability; 5. Others (( please be specific)) |
| Q.39 | Satisfaction of video-based curriculum | 1. Strongly satisfied; 2. Satisfied; 3. Dissatisfied; 4. Strongly dissatisfied |
| Q.40 | Do you want the teacher to be permanent ? | 1. Yes; 2. No; 3. Whatever |
| Q.41 | Preferred to be taught by a teacher | 1. Resident; 2. Attending physician; 3. Associate professor; 4. Professor; 5. Whatever |
| Q.42 | Satisfaction of schedule | 1. Strongly satisfied; 2. Satisfied; 3. Dissatisfied; 4. Strongly dissatisfied |
| Q.43 | Are you willing to recommend other colleagues for refresher training at Fuwai Hospital ? | 1. Strongly recommend; 2. Recommend; 3. Not recommend; 4. Strongly not recommend |
| Q.44 | Satisfaction of on duty schedule | 1. Strongly satisfied; 2. Satisfied; 3. Dissatisfied; 4. Strongly dissatisfied |
| Q.45 | Satisfaction of financial compensation | 1. Strongly satisfied; 2. Satisfied; 3. Dissatisfied; 4. Strongly dissatisfied |
| Q.46 | Reasons for refresher training at Fuwai Hospital (open-ended question) |  |
| Q.47 | Expectations about the content of the study  before the engage in fellow studies (open-ended question) |  |
| Q.48 | Suggestions for managers (open-ended question) |  |
| Q.49 | Suggestions for teachers (open-ended question) |  |
| Q.50 | Suggestions for educational curriculum (open-ended question) |  |
| Q.51 | What do you think is the optimal duration of training? | 1. month; 2. 3 month; 3. 6 month; 4. 12 month; 5. 24 month |
| Q.52 | Regrets perceived at the end of the training (open-ended question) |  |
